# Blood pressure‐related differences in brain health between young African Americans and Caucasian Americans

**DOI:** 10.14814/phy2.14819

**Published:** 2021-03-26

**Authors:** Junyeon Won, Sushant M. Ranadive, Daniel D. Callow, Shuo Chen, J. Carson Smith

**Affiliations:** ^1^ Department of Kinesiology University of Maryland College Park MD USA; ^2^ Program in Neuroscience and Cognitive Science University of Maryland College Park MD USA; ^3^ Department of Epidemiology and Public Health University of Maryland School of Medicine Baltimore MD USA

**Keywords:** blood pressure, cognitive function, cortical thickness, mean arterial pressure, race, younger adults

## Abstract

**Background:**

Although there are moderating effects of race on blood pressure (BP) and brain health in older adults, it is currently unknown if these race‐related differences in cardiovascular and associated brain function are also present in younger adults. The purpose of this study was to investigate the interaction between race and BP on brain health in younger African (AA) and Caucasian Americans (CA).

**Methods:**

We studied 971 younger adults (29.1 ± 3.5 years; 180 AAs and 791 CAs) who volunteered to participate in the Human Connectome Project. Cognitive composite scores, brain volume, and cortical thickness using MRI were cross‐sectionally assessed. ANCOVA was used to examine interactions between race and mean arterial pressure (MAP) on cognitive test scores and brain structure.

**Results:**

After controlling for age, sex, education, and BMI, there were significant Race × MAP interaction effects on cognitive composite scores and cortical thickness. Among AAs but not CAs, as MAP increased, both global cognitive performance and entorhinal cortex (ERC) thickness decreased.

**Conclusions:**

MAP was an important moderator of racial differences in cognitive performance and ERC thickness. Our findings suggest that young AAs may carry a greater hypertension‐associated risk for cognitive brain health deficit. Interventions that address early signs of hypertension in AAs are needed to determine if the racial disparities in BP‐related brain health in late adulthood can be reduced.

## Graphical abstract

Our findings provide evidence that there are significant negative associations between mean arterial pressure and cognitive composite scores and cortical thickness in younger African Americans that were not observed in their Caucasian counterparts. Among African Americans, but not Caucasian Americans, as mean arterial pressure increased, both global cognitive performance and entorhinal cortex thickness decreased. Our findings suggest that young African Americans may carry a greater hypertension‐associated risk for poor cognitive brain health.

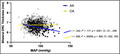

## 1 BACKGROUND

Beyond the well‐documented role of hypertension as a leading risk factor for cardiovascular diseases, the linkage between vascular function and brain health has been extensively studied (Iadecola & Gottesman, 2019). For example, impaired vascular function is associated with cerebral hypoperfusion (Muller et al., 2012), cerebral blood vessel damage (Bohannon et al., 2002), neurovascular inflammation (Morillas et al., 2012), reduced white matter integrity (Faraco & Iadecola, 2013), and smaller brain tissue volumes in regions that are important to cognitive function and memory, such as the frontal cortex and hippocampus (Beauchet et al., 2013). Due to its deleterious impact on brain function, hypertension has been highlighted as an important biomarker to predict future cognitive impairment (e.g., memory, attention, and executive function) (Gorelick & Nyenhuis, 2012; Saxby et al., 2003) and development of dementia (Faraco & Iadecola, 2013).

African Americans (AA) have a disproportionately greater incidence and severity of hypertension and hypertension‐related cognitive impairments than Caucasian Americans (CA) (Redmond et al., 2011). Middle‐aged AAs have been shown to exhibit a faster decline in global cognition, which was associated with higher systolic blood pressure (SBP) and mean arterial pressure (MAP) relative to CAs (Levine et al., 2019). In a neuroimaging study of hypertensive older adults, AAs, compared to their CA counterparts, exhibited impaired network connectivity and cortical thickness of the insula (Chand et al., 2017), a key node of salience network that is important in detecting and filtering salient stimuli and processing information related to emotion, cognition, and reward (Menon & Uddin, 2010). Indeed, higher cumulative blood pressure (BP) over a lifetime, with an onset at a younger age, may be a critical contributor to the faster rate of later‐life cognitive decline among AAs relative to CAs (Levine et al., 2020).

Most of what we currently know about interactions between race and BP on brain health outcomes have come from studies of older and hypertensive individuals. Whether or not the moderating effects of BP on indices of brain function are present in normotensive younger AA and CA adults has not been addressed. Understanding these BP‐related associations with cognitive performance and brain structure in younger AA and CA adults is an essential step toward identifying modifiable factors and implementing early interventions that may help address racial disparities in late‐life cognitive health. Thus, the present study aimed to investigate the moderating role of BP on cognitive function, brain volume, and cortical thickness in younger, normotensive AAs and CAs. Based on evidence showing greater MAP‐related decline in global cognition among middle‐aged AAs relative to CAs (Levine et al., 2019), we hypothesized that negative associations between MAP and cognitive performance, cortical and hippocampal volumes, and cortical thickness will be greater in younger AAs compared to CAs.

## 2 METHODS

### 2.1 Participants

The present study used open‐access data from the University of Minnesota (Wu‐Min) Human Connectome (HC) Project (https://www.humanconnectome.org/study/hcp‐young‐adult/document/1200‐subjects‐data‐release; Van Essen et al., 2013). In short, healthy younger adults were primarily recruited from Missouri. A complete list of eligibility criteria was previously reported (Van Essen et al., 2013). Briefly, exclusion criteria included: (a) Significant history of psychiatry disorder, substance abuse, neurological, or cardiovascular disease; (b) two or more seizures after age 5 or a diagnosis of epilepsy; (c) any genetic disorder (e.g., cystic fibrosis or sick cell disease); (d) Multiple sclerosis, cerebral palsy, brain tumor, or stroke; and (e) Moderate or severe claustrophobia. Self‐reported race was used in this study. Participants provided written consent approved by the Institutional Review Board of Washington University. The entire experimental procedure and data share were performed in compliance with the relevant guidelines and regulations (Glasser et al., 2016). Participants visited Washington University two different days for magnetic resonance imaging (MRI) scanning and BP/cognitive assessments, respectively.

### 2.2 Blood Pressure Measurement, Cognitive Tests, and Mental Health

Prior to the in‐person visit, participants were asked about (a) whether they had ever been told by a physician that they had high BP, and (b) whether they were currently taking medications for high BP. Each participant's resting BP was measured at their visit using established guidelines. Sitting BP in the left arm was measured using a semi‐automatic BP monitor (ADC). A comprehensive battery of neuropsychological tests was administered to evaluate cognitive function. Detailed descriptions about the test administrations and scoring can be found in previous studies (Gur et al., 2010; Weintraub et al., 2013). A comprehensive battery of neuropsychological tests was performed to evaluate cognitive function, including (a) Picture Sequence Memory (to measure episodic memory); (b) Dimensional Change Card Sort (to measure executive function and cognitive flexibility); (c) Flanker (to measure executive function); (d) Oral Reading Recognition Test (to measure reading decoding); (e) picture vocabulary (to measure vocabulary knowledge); (f) Pattern Comparison Test (to measure processing speed); (g) Variable Short Penn Line Orientation Test (VSPLOT; to measure spatial orientation processing); (h) Short Penn Continuous Performance (SPEC; to measure sustained attention); and (i) List Sorting (to measure working memory). Composite scores were calculated as the average of these subtest scores using the NIH Toolbox Cognition Battery Reuben et al., 2013) that included (a) total composite score; (b) crystalized composite score (normalized scores of picture vocabulary and oral reading tests); (c) early cognitive composite score (normalized scores of picture vocabulary, flanker, dimensional change card sort, and picture sequence memory); and (d) cognitive fluid composite score (normalized scores of flanker, dimensional change card sort, picture sequence memory, and list sorting). For the present analysis, we used these four cognitive composite scores as measurements of cognitive performance. Anxiety/depression score assessed as part of life function questionnaire (Achenbach, 2003) in the HC Project was tested for the between‐group difference as a potential covariate affecting BP.

### 2.3 MRI data acquisition

Whole‐brain MRI was conducted using a customized Siemens 3.0 Tesla MR Scanner at Washington University. A 32‐channel head coil was used for radio frequency transmission and reception. A high‐resolution T1‐weighted anatomical image was acquired with gradient echo sequence: field of view =224 mm, voxel size = 0.7 × 0.7 × 0.7 mm, slice thickness = 0.9 mm, repetition time = 2400 ms, echo time = 2.14 ms, inversion time = 1000 ms, flip angle = 8°, and duration = 7:40 min (Van Essen et al., 2012).

### 2.4 MRI data analysis

MRI data were processed using HC Project's optimal processing pipeline (Glasser et al., 2013) where FreeSurfer's (version 5.3.0) automatic cortical reconstruction process for cortical parcellation and subcortical segmentation (recon‐all) (Fischl, 2012) was included. Based on prior evidence documenting the association between hypertension and hippocampal atrophy (Den Heijer et al., 2005; Korf et al., 2004), Freesurfer‐defined bilateral hippocampal volume was chosen *a priori* as region of interest. The interactive effect between race and BP on total gray matter volume was also analyzed as a control to test whether the hypothesized higher BP‐related brain atrophy was driven by the total brain volume rather than bilateral hippocampal volume, specifically.

FreeSurfer's segmentation analysis also quantified cortical thickness by assessing the closest distance between the gray/white boundary and gray/cerebrospinal fluid boundary (Fischl & Dale, 2000). Hypertension is associated with decreased cortical thickness (Seo et al., 2012) and affects brain regions that are vulnerable to aging including entorhinal cortex (ERC) (Raz, 2000). ERC is a brain region where early accumulation of amyloid plaques begins and it later proliferates into the hippocampus (Braak & Braak, 1991). Therefore, bilateral ERC thickness was chosen *a priori* as a region of interest. We also selected bilateral insula *a priori* based on previous evidence suggesting differences in insula thickness between hypertensive AA versus CA older individuals (Chand et al., 2017).

### 2.5 Statistical analyses

We used the Shaprio–Wilk test to determine normality of the demographic data. Between‐group differences (AA vs CA) in demographic characteristics were measured using independent sample *t*‐tests (or Wilcoxon rank sum tests) for continuous variables and a chi‐squared test for the categorical variable. A Shaprio–Wilk test was also performed to determine normality of the cognitive and brain MRI measurements. Due to significant demographic differences between AAs and CAs, between‐group differences in cognitive performance and brain MRI measures were determined using ANCOVA after adjusting age, gender, education, and BMI.

MAP ([(DBP × 2)+SBP]/3) was selected as a measurement of BP as it represents average BP assessed from SBP and DBP. ANCOVA was used to examine the Race × MAP interaction on the cognition and brain structure variables. To accomplish this, race and MAP were first set as independent variables, and age, education, and BMI were added as covariates. Gender was also included as a covariate based on prior investigations showing significant differences in ICV (Raz, 2000; Won et al., 2019) and cortical thickness (Gautam et al., 2013; Savic & Arver, 2014) between men and women. For the volumetric analyses, total intracranial volume estimated by Freesurfer was further included as a covariate. Next, a race by MAP interaction term was added to the model. Cognitive test results and cortical volume and thickness, respectively, were included as dependent variables. Lastly, Benjamini–Hochberg false discovery rate (FDR) correction (Benjamini & Hochberg, 1995) was conducted to control the family‐wise error rate for multiple comparisons in the interaction results. The statistical significance was determined using a two‐tailed alpha = 0.05. All statistical analyses were performed using JASP (version 0.12.2).

## 3 RESULTS

### 3.1 Participants

Detailed study recruitment protocol and retention have been reported previously (Van Essen et al., ,^2013^, ^2012^). Among a total of 1207 participants who completed the study protocol, 126 participants whose race were neither the AA nor CA were excluded. Additionally, 85 participants with Hispanic/Latino or unknown ethnicity were also excluded. Next, 21 participants with missing BP data were excluded and 4 outliers (Cook's distance >0.5) in MAP were further excluded. Finally, cognitive test performance for the remaining 971 participants was analyzed. For the brain volume and cortical thickness assessments, 67 individuals were further excluded due to missing MRI data. Thus, data from a total of 904 participants were included in the brain volume and thickness analyses.

The participants had an average age of 29.1 years, BMI of 27.0 kg/m^2^, and 14.8 years of education. 55.2% of the participants were women. AAs had significantly higher BMI (*t*[967] =5.303, *p *<* *0.0001, *d* = 0.438, 95%CI for *d* = 0.274, 0.601), fewer years of education (t[967] =8.686, *p *<* *0.0001, *d* = 0.717, 95% CI for *d = *0.552, 0.882), and greater MAP (*t*[968] =2.602, *p *=* *0.009, *d* = 0.214, 95%CI for *d* = 0.052, 0.377) compared with CAs. As there was no significant between‐group difference in anxiety/depression symptom score (*t*[965] =0.100, *p *=* *0.920, *d* = 0.008, 95%CI for *d* = −0.153, 0.170), anxiety/depression symptom score was not included as a covariate in the subsequent analyses (see Table 1). None of the participants was taking BP medication.

**TABLE 1:** Demographics and blood pressure data of the participants.

	Total sample (*n* = 971)	African Americans (*n* = 180)	Caucasian Americans (*n* = 791)	Group differences
	Mean (SD)	Mean (SD)	Mean (SD)	*p*‐value (Cohen's *d*)
Demographics				
Age (years)	29.1 (3.5)	28.9 (3.7)	29.1 (3.4)	0.439 (0.064)
Female (n, %)	535 (55.2)	111 (61.6)	424 (53.6)	0.056_F_
BMI (kg/m^2^)	27.0 (5.5)	29.1 (6.5)	26.6 (5.2)	**1.40e−7 (0.217)**
Education (years)	4.8 (1.8)	13.8 (2.0)	15.0 (1.7)	**1.57e−17 (0.717)**
Blood pressure (mm Hg)				
SBP	124.6 (14.4)	126.9 (15.8)	124.1 (14.0)	**0.019 (0.082)**
DBP	77.2 (10.9)	79.0 (11.7)	76.8 (10.7)	**0.015 (0.123)**
MAP	93.0 (11.1)	94.9 (11.9)	92.5 (10.8)	**0.009 (0.103)**
Mental health				
ASR Anxious/Depression Score	53.8 (5.9)	53.8 (5.8)	53.7 (5.9)	0.920 (0.00001)

Notes

_F_ indicates Fisher's exact test; BMI, body mass index; SBP, systolic blood pressure; DBP, diastolic blood pressure; MAP, mean arterial pressure; mmHg, millimeter of mercury; ASR Anxious/Depression Score, measured as part of life function questionnaire (Achenbach, 2003) in the Human Connectome Project; Bold indicates *p* < 0.05.

### 3.2 Between‐group differences on cognitive and brain assessments

After controlling for age, gender, education, and BMI, AAs, compared with CAs, exhibited consistently lower cognitive composite scores, including total composite score (*F*[1,944] =109.081, *p* < 0.0001, *η* ^2^_p_ = 0.104), crystalized composite score (*F*[1,951] =86.583, *p* < 0.0001, *η* ^2^_p_ = 0.083), early cognitive composite score (*F*[1,948] =74.491, *p* < 0.0001, *η* ^2^_p_ = 0.076), and cognitive fluid composite score (*F*[1,944] =62.353, *p* < 0.0001, *η* ^2^_p_ = 0.061]. AAs also had significantly smaller total gray matter volume (*F*[1,895] =22.314, *p* < 0.0001, *η* ^2^_p_ = 0.024), bilateral hippocampal volume (*F*[1,895] =4.729, *p* = 0.029, *η* ^2^_p_ = 0.005), bilateral ERC thickness (*F*[1,896] =33.963, *p* < 0.0001, *η* ^2^_p_ = 0.036), and bilateral insula thickness (*F*[1,896] =18.170, *p* = 0.0006, *η* ^2^_p_ = 0.013] than CAs (see Table 2).

**TABLE 2:** Cognitive test, cortical volume, and thickness data of the participants

	Total sample (*n* = 971)	African Americans (*n* = 180)	Caucasian Americans (*n* = 791)	Group differences
	Mean (SD)	Mean (SD)	Mean (SD)	*p*‐value (η^2^_p_)
Cognitive tests				
Total Cognitive Composite Score	111.6 (20.6)	94.0 (20.4)	115.6 (18.4)	**2.05e−24 (0.104)**
Crystalized Composite Score	108.2 (17.1)	94.2 (18.1)	111.4 (15.2)	**8.97e−20 (0.083)**
Early Cognitive Composite Score	105.6 (16.0)	93.7 (15.9)	108.3 (14.8)	**3.89e−18 (0.076)**
Cognitive Fluid Composite Score	104.9 (17.1)	93.9 (16.7)	107.4 (16.2)	**7.94e−15 (0.061)**
Regional brain volume (mm^3^)				
Total gray matter	686755.6 (67984.6)	639904.3 (60895.5)	697026.7 (65159.3)	**4.52e−6 (0.023)**
Bilateral hippocampus	8913.7 (896.8)	8415.8 (860.9)	9021.7 (868.1)	**0.044 (0.004)**
Cortical thickness (mm)				
Entorhinal cortex	6.7 (0.4)	6.5 (0.4)	6.7 (0.4)	**1.82e−7 (0.030)**
Insula	6.0 (0.2)	5.9 (0.2)	6.0 (0.2)	**8.11e−6 (0.022)**

Notes

η^2^_p_, partial eta‐squared effect size; The group difference analyses were adjusted for age, gender, education, and BMI. Bold indicates *p* < 0.05.

### 3.3 Interaction between race and MAP on cognition

**FIGURE 1:**
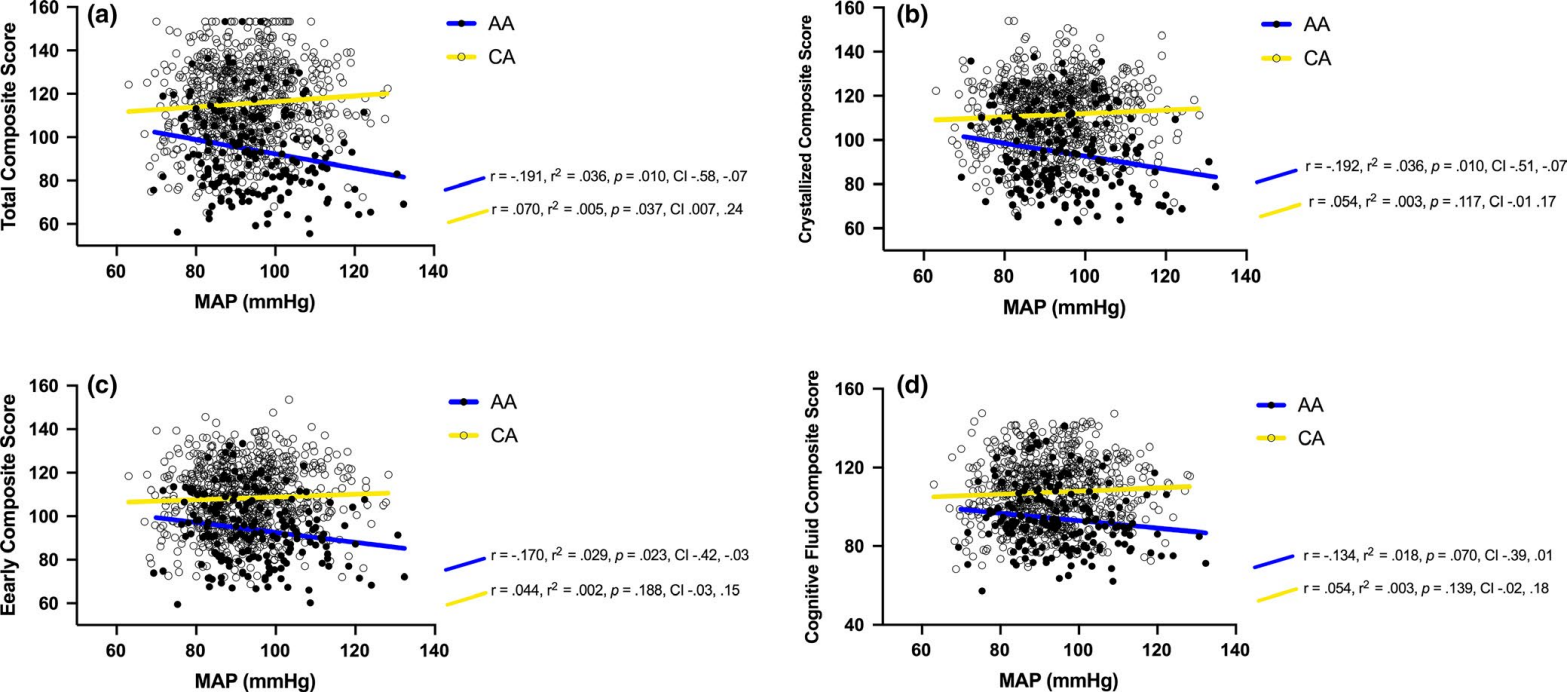
Significant interaction bewteen race and MAP on cognitive task performances (adjusted for age, gender, education, and BMI). The *r*, *r* ^2^ and *p* values indicate the correlation of MAP and cognitive test results for each racial group. The interaction statistical results are presented in Table 3. Notes: MAP, mean arterial pressure; mmHg, millimeters of mercury

Table 3 illustrates the interaction between race and MAP on cognitive composite scores. ANCOVA results revealed significant interactions between race and MAP on total composite score (*F*[1,943] =5.833, *p* = 0.015, *η* ^2^_p_ = 0.006) (Figure 1; Panel a), crystallized composite score (*F*[1,950] =4.324, *p* = 0.037, *η* ^2^_p_ = 0.004] (Figure 1; Panel b), and early cognitive composite score (*F*[1,947] =3.956, *p* = 0.046, *η* ^2^_p_ = 0.004] (Figure 1; Panel c). The interaction between race and MAP on the cognitive fluid composite score was in the same direction but was not statistically significant (*F*[1,943] = 3.548, *p* = 0.059, *η* ^2^_p_ = 0.003] (Figure 1; Panel d). The *p*‐value of the significant effects was greater than FDR correction threshold (FDR‐corrected threshold for total composite score *p* = 0.012, crystallized composite score *p* = 0.025, and early cognitive composite score *p* = 0.037).

**TABLE 3:** Interactions between race and MAP on cognitive test performance, gray matter and hippocampal volume, and entorhinal and insula cortex thickness

	*F* (df)	*p*‐value (η^2^_p_)
Cognitive tests		
Total Cognitive Composite Score	5.833 (1,943)	**0.015 (0.006)**
Crystalized Composite Score	4.324 (1,950)	**0.037 (0.004)**
Early Cognitive Composite Score	3.956 (1,947)	**0.046 (0.004)**
Cognitive Fluid Composite Score	3.548 (1,943)	0.059 (0.003)
Brain volume		
Total Gray Matter	0.00006 (1, 894)	0.993 (0.000)
Bilateral Hippocampus	1.838 (1, 894)	0.175 (0.001)
Cortical thickness		
Entorhinal Cortex	13.669 (1, 894)	**0.0002 (0.015)**
Insula	1.290 (1, 894)	0.256 (0.001)

Notes

df, degrees of freedom, η^2^_p_, partial eta‐squared effect size; The interaction analyses were adjusted for age, gender, education, and BMI. Bold indicates *p* < 0.05.

### 3.4 Interactions between race and MAP on brain volume/cortical thickness

**FIGURE 2:**
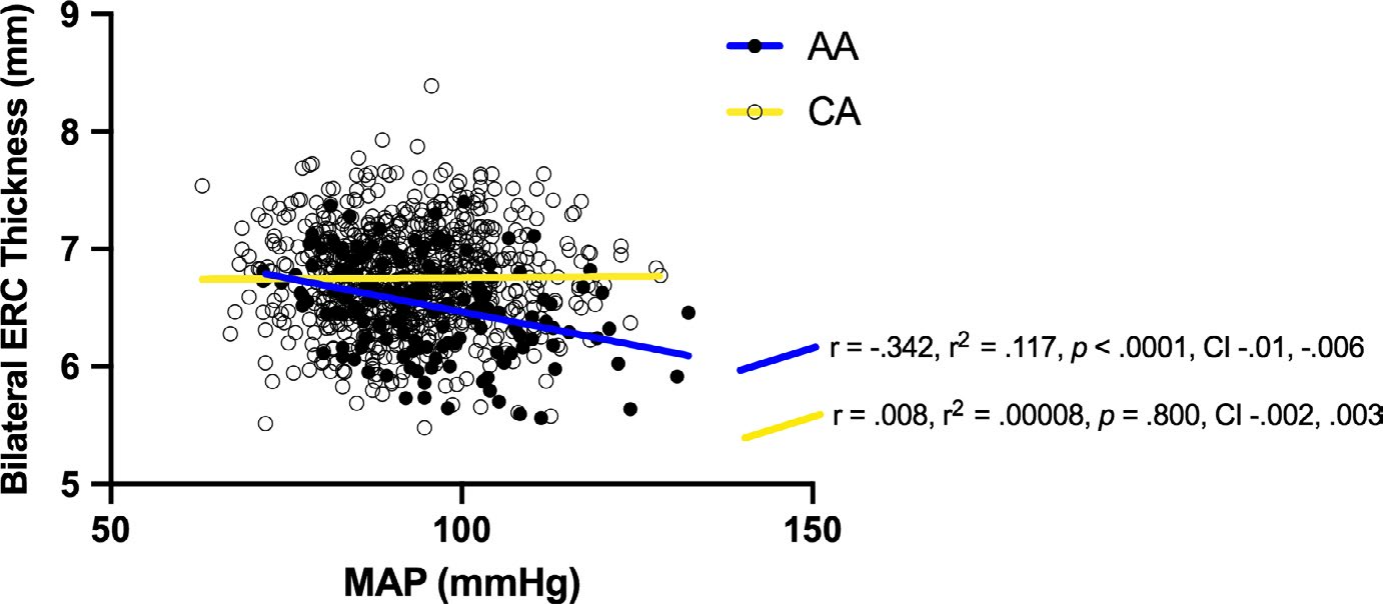
Significant interaction between race and MAP on entorhinal cortex thickness (adjusted for age, gender, education, and BMI). The *r*, *r* ^2^, and *p* values indicate the correlation of MAP and cognitive test results for each racial group. The interaction statistical result is presented in Table 3. Notes: ERC, entorhinal cortex; MAP, mean arterial pressure; mmHg, millimeter of mercury

While there was a significant interactive effect between race and MAP on the bilateral ERC thickness (*F*[1,892] =12.587, *p* = 0.0004, *η* ^2^_p_ = 0.013) (Figure 2), no significant interactions were observed for total gray matter volume (*F*[1,891] =0.00002, *p* = 0.987, *η* ^2^_p_ = 2.92e‐7), bilateral hippocampal volume (*F*[1,891] =1.887, *p* = 0.169, *η* ^2^_p_ = 0.002), and bilateral insula thickness (*F*[1,892] =0.982, *p* = 0.321, *η* ^2^_p_ = 0.001). The *p*‐value of the interaction for ERC thickness was lower than the FDR‐corrected threshold (*p* = 0.012).

### 3.5 Association between ERC thickness and cognitive test results

As the similar Race × MAP interaction patterns were identified in cognitive test results and ERC thickness, we further explored the association between ERC thickness and cognitive test results using multivariable linear regression (adjusted for age, gender, and education). We performed this post‐hoc analysis only in AAs because of the consistent negative associations between MAP and cognitive test outcomes and ERC thickness, which were not observed in CAs (Figures 1 and 2). These analyses in AAs showed that ERC thickness was not significantly associated with total cognitive composite score (*β* = 0.119, *p* = 0.063, 95% CI −0.324, 12.335), crystallized composite score (*β* = 0.057, *p* = 0.344, 95% CI −2.784, 7.930), or early cognitive composite score (*β* = 0.131, *p* = 0.057, 95% CI −0.146, 10.341).

## 4 DISCUSSION

After controlling for age, gender, education, and BMI, our results demonstrate that the markers of brain health between AA and CA younger adults were moderated by BP. As hypothesized, there was a significant Race × MAP interaction on cognitive composite scores and ERC thickness, such that greater MAP was associated with lower cognitive performance and smaller ERC thickness, which was specific to AAs. The current analyses are consistent with, and expand on, previous evidence documenting that MAP‐related associations with lower global cognitive function are stronger in older AAs than their older CA counterparts (Levine et al., 2019). The prior finding utilized the Six‐item Screener Scores (Callahan et al., 2002) as a primary cognitive outcome and three other cognitive tests including Alzheimer's disease word list learning, word list delayed recall, and animal fluency as secondary cognitive outcomes. The present results extend the previous study (Levine et al., 2019) by investigating the significant race by MAP interactions on cognitive composite scores measured using a comprehensive neuropsychological test battery. Although the association between cognitive performance and hypertension in younger adults has been documented (Suhr et al., 2004), the present findings provide novel insight to suggest BP may moderate cognitive performance differently in younger AA compared to CA. Given long‐term exposure to higher BP from young to mid‐adulthood accelerates cognitive decline in midlife (Mahinrad et al., 2020), our findings indicate that the racial disparity in higher BP‐associated cognitive function may begin in early adulthood. While our results should be interpreted with caution as the significant interactions on cognitive outcomes did not survive FDR correction, the consistency of the magnitude and direction of these effects gives confidence that these findings are not merely spurious, and that accepting the null hypothesis would result in a Type II error. Notably, despite the small effect size for these associations, the fact that they were observed in a younger and normotensive cohort has implications for long‐term brain health, as it is plausible that disparity between the AAs and CAs may only get stronger with age.

It has been suggested that the differences in hypertension between the race groups are relevant to a higher prevalence of obesity and cardiovascular diseases in AAs (Pan et al., 2009). In support of these prior findings, we observed AAs had a significantly greater average body mass index (BMI) than CAs. Furthermore, the average BMI (29.1 ± 6.5 kg/m^2^) of AAs was just below the typical cut‐off for the obese category (>30 kg/m^2^). Despite BMI’s significant limitations as an accurate index for body composition (Frankenfield et al., 2001), it provides epidemiological insights on this critical demographic difference between race groups. Another possible physiological contributor to our findings may be genetic differences between race groups related to kidney function. AAs are more likely to experience kidney failure and disease compared to CAs (Harding et al., 2017). Indeed, a recent genome‐wide association meta‐analysis suggested a genetic link between kidney system function and risk of Alzheimer's disease in AAs (Kunkle et al., 2021). Given the strong relationship between kidney function and BP (Young et al., 2002), genetic differences linked with kidney function between the race groups might have been associated with the interaction between race and MAP in the present study. Unfortunately, these genetic and kidney‐related biomarkers are not available in the HC database we analyzed. More importantly, the socio‐economic and/or educational disadvantages (e.g., less access to healthcare, educational and cognitively enriching opportunities) in AAs are also associated and potentially contribute to the racial disparity in development of chronic diseases (Frieden, 2013). Although we have statistically accounted for education, we were unable to adequately address these important socio‐economic determinants in the present study.

Lower insula thickness in older hypertensive in AAs relative to their age‐matched CA counterparts has been documented in a previous study (Chand et al., 2017). In partial agreement with this finding, we have observed lower insula cortex thickness in younger AAs compared to CAs. However, there was no significant interaction between MAP and race on the insula thickness. We also tested the interactive effects on the hippocampus, a brain area crucial for memory, because higher BP is linked with lower hippocampal perfusion (Zola‐Morgan et al., 1986) and reduced hippocampal volume (Beauchet et al., 2013). Higher BP‐related lower hippocampal volume, however, was not detected in the present study. Although there were no significant effects on hippocampal and insula thickness, we discovered a Race × MAP interaction on ERC thickness. One of the earliest changes in neurodegenerative diseases occur within the ERC, and its shrinkage serves as a predictor of future cognitive decline (Du et al., 2001). Age‐related dysfunction in ERC has also been shown to be affected by hypertension (Raz, 2000). Yet, no previous work has corroborated BP‐related differences in ERC thickness patterns between AAs and CAs in older adults. Taken together, these results indicate that BP‐related brain changes may differ by age, such that while the hippocampal and insula may not be impacted by BP in younger adults, higher MAP‐related lower ERC cortical thickness may begin during younger adulthood in AAs. Moreover, although only at borderline statistical significance , there were moderate associations between lower ERC thickness and lower cognitive test scores in AAs, further suggesting that ERC cortical thinning may play a role in the relationship between BP and cognitive performance in younger AA individuals.

### 4.1 Strengths and Limitations

Prior evidence studying older adults used a limited number of cognitive assessments (Levine et al., 2019) or subject cognitive deficit questionnaires (Hajjar et al., 2016) to determine the BP‐related racial disparity on cognitive function. Conversely, the present study employed composite scores from a comprehensive neuropsychological battery to robustly test a wide range of cognitive domains from memory to executive function. We also incorporated the HC Project's high‐resolution neuroimaging data into this analysis to elucidate the possible mechanism deriving BP‐related disparity in cognitive performance between the race groups.

Despite these strengths, there are several limitations to this study that warrant caution when interpreting the results. There was an unequal number of participants between the race groups. However, a bootstrapping analysis provided nearly identical results, suggesting the sample sizes were not an issue. To gain mechanistic insights, future studies need to utilize another physiological measurement such as systemic inflammation, neurotrophic factors/biomarkers, cerebral blood flow regulation, and consider potential genetic influences. Furthermore, beyond BP, future studies need to examine aortic pulse wave velocity which is the gold‐standard technique to investigate arterial stiffness of the large central arteries. The aortic stiffening and associated augmented blood flow pulsatility are closely related to impairment in brain structure and cognitive function (Barnes & Corkery, 2018). Thus, compared to using BP, analyzing aortic pulse wave velocity will advance our understanding of the relationship between vascular structure and brain health. The current study is also subject to the limitations of testing a healthy and normotensive population; thus, younger clinical populations with chronic hypertensive conditions may not demonstrate the significant interactions between race and BP on cognitive and brain measurements found in the present study. Also, since this cross‐sectional design limits interpreting the directionality of the current results, further longitudinal studies are necessary to fully understand the moderating effects of BP on long‐term brain health. Last but not least, we acknowledge that the present investigation is constrained by the HC Project methods and database. Unfortunately, data were not available to explore the social‐cultural factors of race (e.g., income, occupation, zip code, perceived stress, parental support, quality and quantity of education, and exposure to racial discrimination or other traumatic events). Indeed, previous evidence suggests that racial discrimination (Calvin et al., 2003; Coogan et al., 2020) and traumatic events (Edmondson & Känel, 2017; SIVERS GHBAH., 1998) significantly influence the cardiovascular and cognitive health. However, we were not able to account for these factors in our analyses. Nevertheless, we did find that there were no significant differences in depression or anxiety symptoms between AAs and CAs in the present study, suggesting these mental health‐related outcomes may not be associated with the interactions we observed.

## 5 CONCLUSIONS

In conclusion, our findings indicate that a higher MAP is associated with lower cognitive performance in younger AAs, but not in CAs. This suggests that AAs, from early adulthood, may carry a greater risk of BP‐related late‐life cognitive decline. Furthermore, the structural integrity of the ERC, a key region in medial temporal lobe networks related to memory, may be an important mediator or moderator of blood pressure‐related cognitive function in younger AA adults. Although the effects we observed were relatively small, the BP‐related differences in brain health between AAs and CAs were apparent in younger healthy cohort which may accumulate over time and most likely to have long‐term consequences in older people. The present study is limited in its ability to elucidate the underlying social and physiological mechanisms underlying this BP‐related racial disparity. Further efforts are needed to identify these underlying mechanisms and thereby develop practical and effective interventions to mitigate racial differences in cardiovascular and brain health.

## CONFLICT OF INTEREST

The authors have no potential conflicts of interest to report.

## AUTHOR CONTRIBUTIONS

JW, SMR, and JCS conceived and designed the study. JW, SC, and DDC conducted the analyses. JW wrote the paper, and all authors edited the paper.
